# Six-year incidence and some features of cases of brachial plexus injury in a tertiary referral center

**DOI:** 10.4274/tjod.80388

**Published:** 2015-06-15

**Authors:** Meryem Eken, Mehmet Çınar, Taylan Şenol, Enis Özkaya, Ateş Karateke

**Affiliations:** 1 Zeynep Kamil Women and Children’s Health Training and Research Hospital, Clinic of Obstetrics and Gynecology, İstanbul, Turkey; 2 Zekai Tahir Maternity and Womens Health Training and Research Hospital, Clinic of Obstetrics and Gynecology, Ankara, Turkey

**Keywords:** Brachial plexus injury, shoulder dystocia, vaginal delivery

## Abstract

**Objective:**

To present some features and incidence of cases of brachial plexus injury in deliveries at the Department of Obstetrics and Gynecology of Zeynep Kamil Maternity and Children’s Training and Research Hospital, from January 2010 through December 2014.

**Materials and Methods:**

In total, 38.896 deliveries in the Department of Obstetrics and Gynecology of Zeynep Kamil Maternity and Children’s Training and Research Hospital, from January 2010 through December 2014 were screened from a prospectively collected database. We recorded gravidity, parity, body mass index, maternal diabetes, labor induction, gestational age at delivery, operative deliveries, malpresentations, prolonged second stage of deliveries, shoulder dystocies, clavicle and humerus fructures, estimated fetal weight, biparietal diameter, abdominal circumference, femur length, fetal sex, route of delivery, maternal age, and fetal anomalies.

**Results:**

There were 28 (72/100.000) cases of brachial plexus injury among 38.896 deliveries. In the 6-year study period, there were 18.363 deliveries via c-section, whereas 20.533 were vaginal deliveries.

**Conclusion:**

Sonographic fetal weight estimation and clinical examination performed by experienced obstetricians, and active appropriate management of shoulder dystocias seemed to attenuate the incidence of brachial plexus injury in the at risk population in our tertiary referral center.

## INTRODUCTION

Shoulder dystocia is an important cause of neonatal and maternal injury, the incidence of which is reported to be between 0.6-1.4% in vaginal births^(1)^. Shoulder dystocia may result in a major neonatal injuries including brachial plexus palsies, fractures of the clavicle and humerus, hypoxic ischemic encephalopathy, and in rare cases, neonatal death^(1)^.

To overcome this problem, prophylactic ceserean section and health personnel training strategies have been tried for the acute management of shoulder dystocia. Several risk factors have been identified for the selection of appropriate candidates for prohylactic ceserean section; however, none of them reached sufficient sensitivity or specificity^(2,3,4)^. Therefore, to prevent a single case of permanant neonatal injury, very large numbers of prophylactic cesareans are needed, which may result in severe maternal morbidity^(4,5)^. Although training for acute management of shoulder dystocia has been suggested, there has been little objective evidence that this training impacts neonatal and maternal injuries^(5)^. The rate of neonatal brachial plexus injury in the United States and other countries is comparable: 1.5 vs. 1.3 per 1000 total births, respectively. Most of the antepartum or intrapartum factors cannot be used as a guide for selecting patients at high risk for shoulder dystocia with or without brachial plexus injury^(6)^. Previous data showed sequential use of vacuum and forceps to be risk a factor for both neonatal and maternal injury^(7)^. Data suggest that the McRoberts’ maneuver is adequately successful at relieving shoulder dystocia in the majority of cases and may be associated with decreased morbidity compared with other maneuvers. As a consequence, the McRoberts’ maneuver is recommended as the initial technique for disimpaction of the anterior shoulder^(8)^.

The aim of this study was to present some features and incidence of cases of brachial plexus injury in deliveries at the Department of Obstetrics and Gynecology of Zeynep Kamil Maternity and Children’s Training and Research Hospital, from January 2010 through December 2014.

## MATERIALS AND METHODS

At the Zeynep Kamil Women and Children’s Health Training and Research Hospital, prospectively collected records of labor and delivery, nursery, and neonatal intensive care unit were reviewed and identified from the computer database of all deliveries that included details of the labor, all cases of Erb’s/Duchenne and Klumpke’s palsies and brachial plexus injury confirmed by a pediatric neurologist’s examination. Among all cases of brachial plexus injury, we recorded gravidity, parity, body mass index, maternal diabetes, labor induction, gestational age at delivery, operative deliveries, malpresentations, prolonged second stage of deliveries, shoulder dystocias, clavicle and humerus fructures, estimated fetal weight, biparietal diameter, abdominal circumference, femur length, fetal sex, route of delivery, maternal age, and fetal anomalies. After delivery of the fetal head, shoulder dystocia was considered in cases when there was need for additional obstetric maneuvers in addition to gentle downward traction. Maneuvers used after unsuccessful head traction were identified as follows: McRoberts maneuver, suprapubic pressure, Rubin maneuver, delivery of the posterior shoulder, Woods corkscrew maneuver, Gaskin maneuver (delivery in the maternal knee-chest position), Zavanelli maneuver, and fundal pressure. Prolonged second stage of labor was defined as longer than 2 h and was extended to more than 3 h when regional analgesia was used in nulliparas. For multiparas, 1 h was the limit, but was extended to 2 h with regional analgesia based on deviations from Friedman’s curve (Macrosomia was defined as a birth weight equal to or more than 4000 g^(9)^. Estimated fetal weight deviation was defined as the difference from expected fetal weight for gestational age, expressed as a percentage)(estimated weight) expected weight)/(expected weight) *100)^(10)^. Body mass index (BMI) was calculated (initial weight/height2 (kg/m^2^). Excessive weight gain during pregnancy was defined as weight gain above 16 kg between the first visit and delivery. Head circumference was measured on a transverse view of the fetal head in an axial plane at the level at which the continuous midline echo was broken by the cavum septi pellucidi in the anterior third and derived from measurement of the occipitofrontal diameter and biparietal diameter^(11)^. Abdominal circumference was measured on a transverse plane, just above the level of the cord insertion and computed from orthogonal diameters^(12)^. To measure femur length, a sonographic plane was obtained including the entire femoral diaphysis, with both ends clearly visible and at an angle of  <45° to the horizontal^(13)^. Estimated fetal weight was calculated in all cases using the following formula of Hadlock et al.^(14)^. Data was entered into SPSS version 15. Some clinical and demographic characteristics of the study population were summarized using descriptive statistics. Chi-square cross tables were used to summarize data of categorical variables. P<0.05 was accepted as statistically significant.

## RESULTS

There were 28 (72/100.000) cases of brahial plexus injury among 38.896 deliveries. Some 18.363 (47.21%) of the deliveries were via c-section, whereas there were 20.533 vaginal deliveries in the 6-year study period. Some demographic and clinical parameters of the cases of brachial plexus injury are summarized in Table 1. The data of women who were grouped according to the WHO BMI classification are summarized in Table 2. Some features of the groups summarized in Table 3.

## DISCUSSION

In this article, we wanted to point out some clinical characteristics of obstetric cases of brachial plexus injury that occurred in our tertiary referral center. Analyses of the data generated from 2010 through 2014 showed that the incidence of obstetric brachial plexus injury was lower our center, which is thought to be the result of increased awareness, experience, and training. Another explanation for this decrement is the increasing rates of ceserean section that has resulted from the increased awareness of parents of the responsibilities of health workers, which can lead to litigation. In the literature, the incidence of brachial plexus injury has been reported to be 1-2 per 1000 births^(15)^. The incidence of obstetric brachial plexus injury in our study population was 0.72 per 1000 births including both vaginal and ceserean deliveries. Shoulder dystocia and brachial plexus injury have an unpredictable nature. A study on risk factors for brachial plexus injury showed that shoulder dystocia, macrosomia, labor dystocia, vacuum delivery and vaginal breech deliveries were significant risk factors for neonatal brachial plexus paralysis. The study found no association between maternal characteristics such as obesity and diabetes. The authors concluded that despite the improved knowledge of risk factors associated with brachial plexus paralysis, unfortunately, this condition cannot be predicted or prevented^(16)^. Moreover, other data showed no reliable factors associated with the brachial plexus palsy^(17)^. The overall incidence of neonatal brachial plexus palsy, both transient and persistent impairment, was reported to be 1.5 per 1000 total births in the American Congress Obstetricians Gynecologists bulletin in 2014. Due to the changes of health policies in Turkey and with increasing health litigations since 2002, ceserean section rates have started to increase. The increased rate of ceserean deliveries (47.21%) is thought to be the major determinant of this low incidence of injury in our population, but we should not disregard the role of trained health workers and well-conducted maneuvers during shoulder dystocia management. In a previous study, direct fetal manipulation techniques used to overcome shoulder dystocia were not found to be associated with an increased rate of brachial plexus injury^(18)^. A study conducted in 1997 concluded that the McRoberts’ maneuver was associated with a significant degree of success in relieving shoulder dystocia and may be associated with decreased morbidity compared with other maneuvers^(19)^. Although the Cochrane review concluded that prophylactic maneuvers should not be used to prevent shoulder dystocia, if a recognizable risk factor for shoulder distocia is present in our institution, a prophylactic McRoberts maneuver is used most of the time^(20)^. When the role of sonography is considered in the prevention neonatal injury, a review published in 2004 reported that the true value of ultrasonography in the management of fetal macrosomia may be its ability to rule out the diagnosis. Ultrasound-derived fetal weight estimates alone are not sensitive or specific enough to determine a route of delivery^(21)^. However, since then the quality of sonography equipment and experience in this area has greatly developed. A recently published study concluded that the impact of diabetes as a risk factor has been minimized by the means of improved screening and treatment, and antenatal sonography was thought to be a promising tool; however, its predictive value is still too low to be used alone^(22)^. In conclusion, sonographic fetal weight estimation and clinical examination performed by experienced obstetricians, and active appropriate management of shoulder dystocias with increased rates of ceserean section seemed to attenuate the incidence of brachial plexus injury in the at risk population at our tertiary referral center.

## Figures and Tables

**Table 1:**
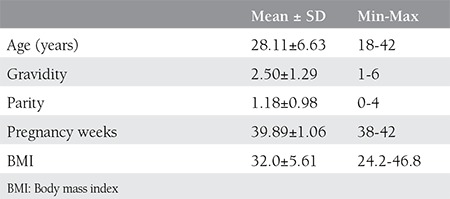
Maternal demographic characteristics

**Table 2:**
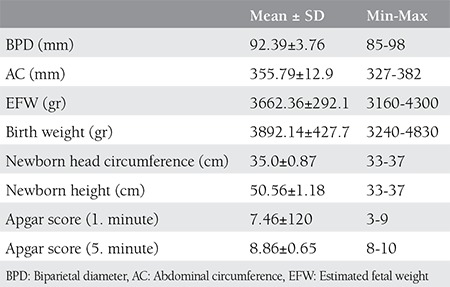
Fetal and neonatal characteristics

**Table 3:**
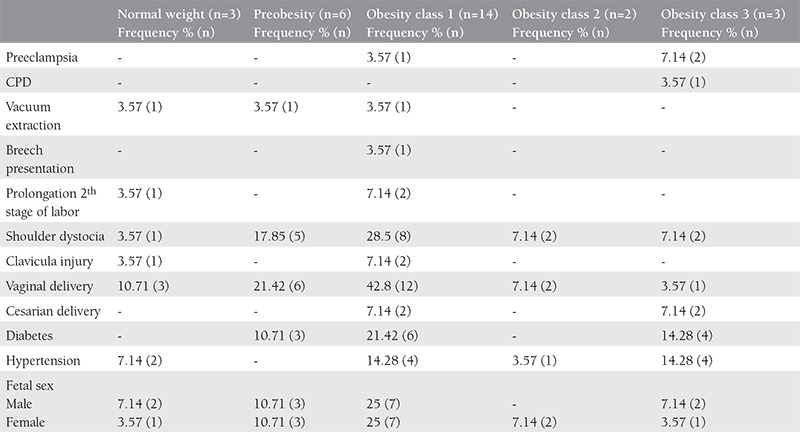
Some characteristics of groups established relative to body mass index
